# *Malassezia* in Inflammatory Bowel Disease: Accomplice of Evoking Tumorigenesis

**DOI:** 10.3389/fimmu.2022.846469

**Published:** 2022-03-04

**Authors:** Qiyu Yang, Jing Ouyang, Damao Pi, Li Feng, Jiadan Yang

**Affiliations:** ^1^ Department of Radiation Oncology, Chongqing University Cancer Hospital & Chongqing Cancer Institute & Chongqing Cancer Hospital, Chongqing, China; ^2^ Chongqing Public Health Medical Center, Chongqing, China; ^3^ Department of Pharmacy, The First Affiliated Hospital of Chongqing Medical University, Chongqing, China; ^4^ Department of Pharmacy, The First Affiliated Hospital of Chongqing Medical and Pharmaceutical College, Chongqing, China

**Keywords:** *Malassezia*, fungus, inflammation, inflammatory bowel disease, cancer

## Abstract

Accumulating evidence indicates that patients with inflammatory bowel disease (IBD) have a significantly higher risk of developing different cancers, while the exact mechanism involved is not yet fully understood. *Malassezia* is a lipid-dependent opportunistic yeast, which colonizes on mammalian skin and internal organs. Also, dysbiosis in fungal communities accompanied by high level of *Malassezia* are fairly common in inflammatory diseases such as IBD and various cancers. In cancer patients, higher levels of *Malassezia* are associated with worse prognosis. Once it is ablated in tumor-bearing mice, their prognostic conditions will be improved. Moreover, *Malassezia* manifests multiple proinflammatory biological properties, such as destruction of epithelial barrier, enrichment of inflammatory factors, and degradation of extracellular matrix (ECM), all of which have been reported to contribute to tumor initiation and malignant progression. Based on these facts, we hypothesize that high levels of *Malassezia* together with mycobiome dysbiosis in patients with IBD, would aggravate the microecological imbalance, worsen the inflammatory response, and further promote tumorigenesis and deterioration. Herein, we will discuss the detrimental properties of *Malassezia* and explore the key role of this fungus in the correlation between IBD and cancer, in order to take early surveillance and intervention to minimize the cancer risk in individuals with IBD.

## Introduction

Inflammatory bowel disease (IBD) belongs to chronic idiopathic gastrointestinal (GI) inflammatory diseases, characterized by imbalance of the intestinal microbiome (1). Typically, IBD contains Crohn’s disease (CD) and ulcerative colitis (UC), of which the common characteristic is the inflammation in the GI wall. Their main distinction is the site and depth of lesions. UC is generally limited to the colon, while CD may include the whole intestine ranging from the mouth to the rectum (2). The rapid pace of IBD expansion over only several decades is disturbing, which has led to serious socioeconomic burden (3).

Emerging evidence suggests that the damaged intestine barrier and gut microbiome dysbiosis are closely linked with the genesis of IBD (4–6). Generally, the batches of bacteria, viruses, archaea, fungi, and eukaryotic microbes inhabiting in the GI tract are referred as gut microbiota, which have already formed a mutually beneficial correlation with the host (7, 8). Among the collection of bacteria, persistent enteric pathogens including adhesion-invasive *Escherichia coli* (AIEC) and *Clostridium difficile*, may act as a trigger for IBD (6). Besides bacterial contribution, there is increasing awareness regarding the impact of mycobiome as immunologically reactive components. On the whole, IBD and ordinary population differ in mycobiome. Alterations in gut mycobiome as well as their communications with other intestine microbiota are essential to maintain the intestinal barrier, such as in IBD (9–14). Reports showed that higher abundance of *Candida albicans* was observed in the intestinal tract of UC patients, when compared to controls, conversely, the decreased amount of *C. albicans* indicated the improvement and recovery of UC (12). Studies also confirmed that when compared with healthy people, one specific commensal fungi *Malassezia* yeast has been identified particularly abundant in people with IBD, which may scale up inflammatory cytokine production and aggravate inflammation in IBD (10, 11, 13, 14). In addition, when the microflora balance is disturbed or the host immune defense is impaired, fungi may spread or transfer from the original symbiotic habitat to other important organs such as the gut, thus becoming a susceptible factor for life-threatening infection (15). The mouse model of psoriasis showed that pre-exposure of symbiotic fungus may significantly worsen tissue inflammation through enhancing T helper type 17 (Th17)-dependent immune responses and phagocytosis of neutrophils (16).

Recently, it has been noticed that malignancies are prevalent in people with IBD (17–20). Reports showed that standardized incidence ratios (SIRs) for pancreatic cancer (PC) in IBD cases were 7.6-fold higher than the ordinary persons (21). For colorectal cancer (CRC), when compared with the reference individuals, the cancer risk in patients with IBD increased considerably, up to around 10 times (22, 23). Studies indicated a strong association between IBD and bile duct cancer, and the SIR could rise remarkably in patients with UC for intrahepatic cholangiocarcinoma (24, 25). Extraintestinal analyses also revealed that the IBD group had a significantly elevated risk for skin cancer (e.g., melanoma), particularly among CD patients or elder population (26–28). Furthermore, evidence has demonstrated that inflammation acts a substantial role in oncogenesis (29, 30).

However, the underlying mechanism linking IBD to cancer remains to be further clarified. *Malassezia*, as representative fungal commensals, may be the key to push aside this dense fog. Based on this, this work is intended to discuss and elaborate the interplay between *Malassezia*, IBD, and cancer.

## Hypothesis

**Figure 1:**
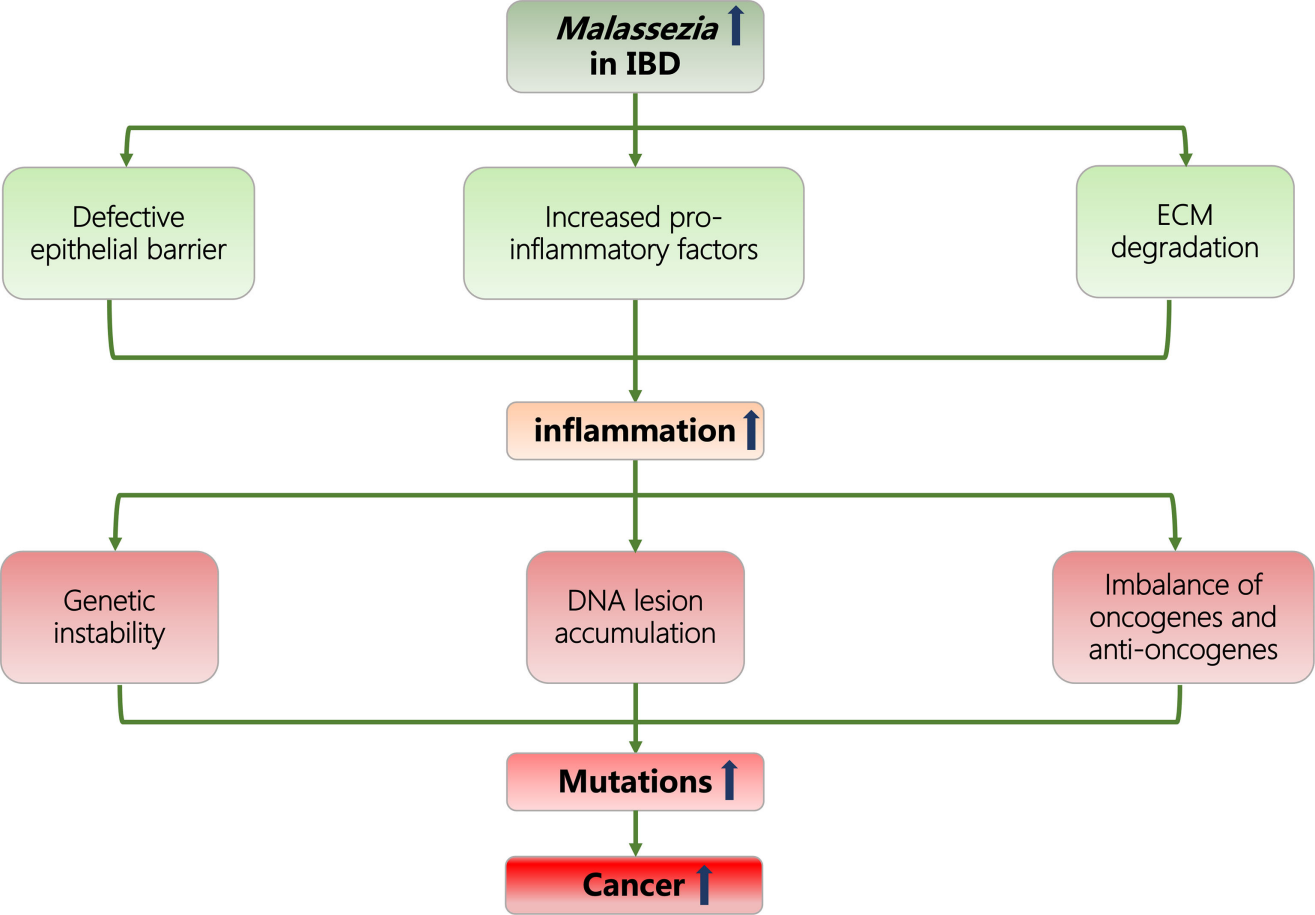
Flowcharts representing the association of *Malassezia*, IBD, inflammation, and cancer. IBD, inflammatory bowel disease; ECM, extracellular matrix.

Considering current evidence, we hypothesize that the particular enrichment of *Malassezia* genius in the gut microbiome could promote inflammatory responses in IBD patients through the following microbiological characteristics: disrupting the integrity of epithelial barrier; increasing the release of proinflammatory molecules; and degrading the extracellular matrix (ECM). In this manner, *Malassezia* can further induce neoplasia and raise cancer incidence in the IBD population under inflammatory conditions (**Figure 1**).

## Life and Properties of *Malassezia*

### General Properties of *Malassezia*

*Malassezia* pertains to the category of lipid-dependent yeast and is an essential symbiotic organism resident on the mammalian skin, hair, and GI tract (31). Under certain scenario, *Malassezia* spp. may switch to opportunistic pathogens.

When referring to microbial variability in different body sites, the skin microbiome is known for its relatively high fungal presence (32). *Malassezia* is the dominated eukaryotic component of microbial communities identified in human and is most abundant in craniofacial sebaceous glands and relatively low in arms or trunk, due to the enrichment of the lipid nutritional sources (33, 34). So far, as many as 20 species of *Malassezia* strains have been identified (35, 36). *Malassezia restricta* and *Malassezia globosa* are the top 2 most abundant in human skin (37). However, the distribution of *Malassezia* is not confined to the skin, high-throughput sequencing revealed that they are also detected at a relatively high frequency in the GI and respiratory tract sample sets, with positive rates of 88% and 86%, respectively (38, 39).

Shaping on fungal community is changing with age. The skin microbiota of neonates delivered vaginally resembled their mother’s vaginal mycobiome, while that of neonates born through cesarean section was similar to the skin surface of mothers (40, 41). *Malassezia* colonized once after birth and was more resembling adult microbiome assemblage, as driven by maternal hormones (42, 43). The relative abundance of *Malassezia* in infant skin was only 2%, while cesarean-born infants owned lower (44). The sebaceous gland entered a dormant state within 6 months after birth, thus *Malassezia* returned to low enrichment. *M. globosa* predominately colonized on prepubertal skin. During adolescence, the increase of lipid levels in the sebaceous glands can lead to a simultaneously increased percentage with *Malassezia* (45). Skin fungal community analysis showed that *Malassezia* fungus was absolutely dominant in adults, with predominance up to over 90%. In contrast, *Malassezia* was relatively lower in children under 14, but the fungal community was more diverse (46).

### Pathogenic Properties of *Malassezia*

Studies showed that *Malassezia* spp. were associated with numerous inflammatory diseases, such as dermal inflammation (16, 47), IBD (13, 14), CRC (48), pancreatic carcinoma (31), and severe infections (49). In general, *Malassezia* is mainly colonized in the skin (50). However, the availability of lipid nutrition within the GI tract may facilitate the localization and survival of *Malassezia* in the gut, and its nutrition can be accessed from host diet or intestinal fungal synthesis (51, 52). It is speculated that *Malassezia* might immigrate to the GI tract along with the diet; however, solid evidence is lacking. Manifold factors can influence the pathogenicity of *Malassezia*, which involves the virulence and quantity of *Malassezia*, environmental conditions including humidity, temperature, oxygen, and fatty acid nutrients, as well as the susceptibility of the host (53). So far, several viewpoints have been proposed to reveal the pathogenic behaviors of *Malassezia*, which may bring more dawn for understanding the inflammatory or infectious pathogenesis.

#### Epithelial Barrier Defects

The epithelial barrier is organized as a protective and complicated system that allows nutrient exchange while preventing the displacement of microorganisms and their metabolites. This tight barrier is obviously a main hurdle which must be broken through for microbial antigens to enter the human body, for example, in the lumen of the gut (54). *Malassezia* could produce increased irritating free fatty acids through metabolism, typified by oleic acid (OA), which may damage the permeability of the skin barrier and lead to skin itching or even exfoliation (55, 56). Because of this, when applying OA to the scalp, it would induce scalp flaking in patients with seborrheic dermatitis (55). *Malassezia* species were kind of lacking synthase genes for fatty acid, which may be supplemented by enhancing the expression of genes encoding secretory hydrolases in *Malassezia* genome to generate fatty acid (57). Among them, the extracellular lipases and phospholipases secreted by *Malassezia* could severally influence its virulence factors on the release of distinct metabolites and the cell wall characteristics itself, in order to facilitate epithelium targeting, lesion aggravation, and barrier disruption (57, 58). It is reported that the lipase activity of *Malassezia* is linked to the pathogenesis of inflammatory skin diseases *in vitro* (59). *Malassezia* phospholipase activity has also been reported to be related to its virulence in dogs (60). What is more, other environmental determinants such as increased epidermal water loss, loss of tight junction proteins, decrease in both cholesterol and free fatty acid, and high pH value, could exhibit catalytic effects in the pathogenesis of *Malassezia*, thus inducing and exacerbating cutaneous inflammation (61, 62). Defective cutaneous barriers failed to provide adequate protection against microbes or allergens, instead they may assist *Malassezia* to enter the blood circulation system, resulting in immune activation and inflammatory process (63).

We own innate immunity in our genomes which provides a defense against *Malassezia* infection. As a positive regulator, mast cells (MC) can detect and control the fungi *Malassezia* at the infected site, which may be activated by Toll-like receptor 2 (TLR2) (64, 65). Macrophages can effectively defend the host against the attack of opportunistic fungal pathogens through phagocytosis and collection of phagocytic contents, affected by TLR9 (66, 67). As innate immune receptors, Dectin-2 and Mincle were mainly involved in the immune recognition to *Malassezia*, arousing the production of pro- and anti-inflammatory factors (68). Moreover, *Malassezia* may induce a reciprocal activation between natural killer (NK) cells and dendritic cells (DCs), in which NK cells would promote the maturation and costimulatory capacity of DCs, as well as accelerate the release of interleukin-8 (IL-8) in DCs (69, 70). These innate immune responses will play an important role in the subsequent adaptive immunity including activation of T cells. *Malassezia* may also promote the progression of inflammation directly or indirectly by mediating host receptor recognition, promoting the release of proinflammatory cytokines and secreting chemicals or vesicles. Within the host, C-type lectin receptor (CLR) family could specifically recognize fungal microorganisms and initiate adaptive immune responses (68, 71). CLRs were specialized in sensing the carbohydrates in the cell wall of *Malassezia*, mediating the signaling adaptor caspase recruitment domain-containing protein (CARD)-9 to drive the polarization of CD4+ T lymphocytes into IL-17-producing immune cells, such as γδ T cells or innate lymphoid cells (ILCs) (71, 72). *Malassezia* could generate indoles, the ligands for the aryl hydrocarbon receptor (AhR), which may also promote Th17 differentiation and IL-17 secretion through activation of AhR signaling (73, 74). By eliciting the production of inflammatory cytokine IL-17 strongly, *Malassezia* spp. has been reported to stimulate tissue inflammation, destroy skin integrity, and further contribute to or augment epicutaneous infections such as atopic dermatitis (AD) in murine models (72). Other proinflammatory cytokines including IL-18, IL-8, and IL-6 and Th22 chemokines including C-C Motif Chemokine Ligand 17 (CCL17) were significantly increased after exposure to *Malassezia* as well, so as to worsen local inflammation (75, 76). It has been confirmed that, compared with the negative group, *Malassezia* colonization can induce CARD9-S12N polymorphism and strongly enhance the release of cytokines such as IL-10 or tumor necrosis factor alpha (TNF-α) in either wild-type (WT) or *Card9* ^−/−^ colitis mice (13). Moreover, *Malassezia* was able to activate NLRP3 inflammasome through Dectin2/CARD9 signal, accelerating the generation of IL-1β to aggravate inflammation (77). Zhang et al. also demonstrated that *Malassezia* can produce nanovesicles rich in allergens or proteins, which may initiate and maintain inflammation by activating the nuclear factor-κB (NF-κB) pathway and upregulate IL-6 production in the immune microenvironment (78).

#### ECM Degradation

ECM is a highly dynamic acellular network composed of collagen, fibronectin, and several other proteins (79). It is of great importance in the inflammatory process. Reports showed that ECM was involved in the signal transmission to recruit inflammatory cells, stimulate cell migration, and restore inner homeostasis for coping with external stimulus (80). Variation or degradation of ECM components has been confirmed to have a close relationship with the progression of various inflammatory diseases, such as AD (81, 82). Some virulence factors including acid sphingomyelinase and aspartate protease, which were secreted by *Malassezia*, could mediate ECM degradation and participate in the pathogenesis (83). MgSAP1, one unique secreted *M. globosa* protease, has been implicated in hydrolyzing host proteins to provide nutrition and destroy ECM elements, so as to facilitate pathogen adhesion in the inflamed areas (84). Adhesion is a decisive step in the pathogenesis of microbial infection (85). One close homolog of secretory MgSAP1 protease produced by *Malassezia furfur* is MfSAP1, which owned high catalytic efficiency in extracellular proteins of human skin, particularly when substrate collagen was thermally denatured (86, 87). It has been proved that MfSAP1 was likely to modify the epidermal and dermal environment through degrading key components of skin-correlated ECM, such as vitronectin, fibronectin, and thrombospondin, even at low proportions of enzyme to the substratum. Accordingly, high concentrations of MfSAP1 could rapidly and sensitively cleave these ECM proteins and inhibit cell migration and attachment to the fundamental ECM, thus attenuating re-epithelization process and retarding cutaneous wound healing in an acute traumatic cell model (86).

Apart from proinflammatory motivation, *Malassezia* has manifested some other biological features, including unique cell-defense characteristics. *Malassezia* owns quite thick and unique multilayered cell walls, which could protect themselves from complex environmental stress and help to escape phagocytosis (88). In particular, *Malassezia* was able to form biofilms on their surface, which was correlated to the emergence of drug resistance and the maintenance of virulence (89). Beyond these described circumstances, *Malassezia* may promote disease progression by modulating the pathogenicity of other microorganisms, for instance *Staphylococcus aureus* through microbial communications (90).

## Increased *Malassezia* and Its Relevance With Inflammation in IBD and Other Inflammatory Diseases

In recent years, the detection of *Malassezia* in the digestive tract has attracted public attention. Microbial diversity analysis showed that Basidiomycota phyla ranked as second major dominators, following Ascomycota phyla, within gut fungal microbiota in both healthy controls and IBD patients, although there were some variations among different disease phenotypes (14). As contrasted to healthy persons, *Malassezia* genera were observed with a high prevalence in people with IBD, which was one primary reason to the increase of Basidiomycetes in IBD (11, 14). Sokol and his team revealed that the higher abundance of *Malassezia* was noticed in the acute stage of IBD population (14). In one retrospective cohort, when compared with healthy volunteers, increased abundance of *Malassezia* was also detected in patients with IBD (50). Limon and his colleagues reported that *Malassezia* species, represented by *M. restricta*, were more enriched in the sigmoid colon mucosa of CD patients, where massive monocyte-derived DCs were involved in the following pathogenesis (13). Statistic data suggested that *M. sympodialis* and its extract were able to initiate MC to secret cysteinyl leukotrienes and intensify IgE-dependent immunoreactions *in vitro*, which might possibly lead to deteriorative inflammation in IBD as well (91, 92). The polymorphism of CARD-9 in CD would encourage the colonization of *M. restricta* yeast in the intestine, which may worsen or even exacerbate the intestinal inflammation by strongly evoking proinflammatory responses of macrophages and monocyte-derived DCs in murine models (13). Beyond this, Liguori et al. also demonstrated that the overall load of *Malassezia* in CD patients was significantly increased in contrast to ordinary ones (*p* < 0.05), suggesting that *Malassezia* may play a role in the pathogenesis of mucositis (11). Similar results have been obtained from the clinical analysis on UC. Regarding fecal fungal composition, there was a significant difference in only several fungal genera between UC patients and non-IBD controls, among which *Malassezia* owned a relatively higher abundance in patients with UC, particularly when at the active phase (93).

Aside from IBD, evidence suggests that the enrichment of *Malassezia* is also obviously increased in other chronic or inflammatory diseases, such as psoriasis, AD, and cystic fibrosis (CF). Scholars found out that the average quantity and species diversity of *Malassezia* in psoriatic patients were higher than those in healthy individuals, particularly in skin lesions (94). *Malassezia*-positive culture rate of scalp skin samples was 85% and 50% in patients with psoriasis and healthy subjects, respectively (95). The more serious the lesion, the higher is the positive rate. Studies also indicated that *Malassezia* allergens could induce immunoglobulin E (IgE)-mediated sensitization in AD subjects (96). There was a significant positive correlation between *Malassezia*-specific IgE levels and AD severity (97). Mittermann and colleagues reported that compared with healthy controls, *Malassezia* was more abundantly detected in sputum specimens of asthmatic patients, particularly in children (98). High detection rates of *Malassezia* were observed in respiratory tract of CF patients as well (99, 100). Beyond this, higher *Malassezia* colonization was detected in subjects with neurodegenerative diseases, such as Alzheimer’s disease, Parkinson’s disease, and amyotrophic lateral sclerosis (101–103). In most cases, the severity of the mentioned diseases is positively correlated with the abundance of *Malassezia* detected.

It seems ubiquitous that the appearance frequency of *Malassezia* yeast differs in distinct human body site. As *Malassezia* strains are resident members of cutaneous mycobiome, it is not so esoteric to understand the relationship between the high prevalence of *Malassezia* and psoriasis or AD. Apart from this, *Malassezia* may be an overlooked fungal pathogen that is neglected to participate in inflammatory splanchnic diseases, particularly in IBD.

## Roles of *Malassezia* and Inflammation in the Context of Cancer

### *Malassezia* Is Associated With Cancer

Host–microflora interactions in the tumor microenvironment play an important driving role in tumorigenesis and progression. In various noninfectious diseases such as cancer, the emphasis of most research is concentrated in bacteriome. Recently, scholars have recognized the significance of mycobiome and microbial dysbiosis in genesis and development of neoplasms. Since its powerful potential in cancer development, the role of one cardinal fungus genus *Malassezia* has just begun to come under scrutiny.

Epidemiological data suggested that abundant yeast niches of *Malassezia* were overlapping with cancerous regions of basal cell carcinoma in humans and animals such as dogs or cats (96, 104). This yeast may promote dermal carcinogenesis through synthesis and activation of AhR ligands and further inhibition of cell caducity (104). Moreover, Aykut and his team reported that *Malassezia* was remarkably elevated in the pancreas of PC patients and mice models than that in healthy volunteers. Furthermore, they have demonstrated that repopulation with *Malassezia* could promote PC through mannose-binding lectin (MBL) signal pathway, thus accordingly its ablation with amphotericin B in murine models was found to slow oncogenic progression (31, 105). *Malassezia* also showed higher relative abundance in patients with oral squamous cell carcinoma (OSCC), compared with control volunteers (106). In addition, Gao and collaborators discovered that although there was no significant difference in stool mycobiota diversity between CRC patients and healthy controls, the fungal subgroup *Malassezia* genus was more enriched in people with CRC, which was positively correlated with tumor progression (48). Similar results were obtained in another analysis performed by Coker and coworkers. They found that when compared with control subjects, *Malassezia* strains were elevated significantly and the mycobiome diversity was specifically modified in CRC (107). This may provide a potentially predictive diagnostic marker for CRC. However, so far, there is no exact hypothesis about the underlying mechanism of *Malassezia* involved in cancer, and the relationship between *Malassezia* and CRC still awaits further study.

### Inflammation and Cancer

There are many hypotheses about the pathogenesis of tumors, among which the theory of inflammatory mechanism is the widely accepted one. Usually, inflammation is fundamental to fight against harmful or pathogenic stimuli, hasten the wound to restore and maintain normal function of tissues, which involves in endothelial cells, immune cells, and inflammatory agents (108). Self-limited acute inflammation is beneficial in the healing process (109). However, when it is out of control, it may develop into chronic inflammation, induce tissue lesions and predispose to cancer (110), including tumorigenesis, progression, and metastasis (111). Only a small portion of cancers are ascribed to germ line mutations, while 90% of cancers are associated with somatic mutations and environmental hazards, and the latter is always linked to chronic inflammation or infections (112). Epidemiological investigations showed that inflammation was closely related to the occurrence of about 20% of all cancers (113). Triggers of chronic inflammation for enhanced risk or progression of cancer include microbial infections, such as *Helicobacter pylori* in gastric adenocarcinoma and chronic hepatitis B virus (HBV) in hepatocellular carcinoma (HCC), and inflammatory diseases (e.g., UC in CRC), which were closely bound up with genetic instability (114–116). Accordingly, with *H. pylori* being eradicated, the conditions of atrophic gastritis and intestinal metaplasia would be improved or eliminated, thus potentially suppressing the generation of gastric cancer (117). Moreover, existing evidence has shown that hypoxia-associated inflammatory cytokines or chemokines were significantly elevated in the tumor microenvironment, for example, IL-6, IL-1, and TNF (29, 118). Anti-inflammatory drugs may benefit cancer patients, such as TNF blockade and nonsteroidal anti-inflammatory drugs (NSAIDs) (119, 120).

### *Malassezia*-Related Inflammation Contribute to Tumorigenesis

As mentioned above, *Malassezia* has exhibited profound proinflammatory effects and been linked to oncogenesis. On the other hand, inflammation can influence all the stages of tumorigenesis and drive tumor development and metastasis. Based on prior elaboration, *Malassezia*-associated inflammation may promote tumor initiation and malignant progression through multiple approaches:

(1) Barrier impairment—epithelial barrier deterioration, including aberrant production of mucin and defective expression or organization of tight junctional proteins, induced by oncogene activation, was an early malignant behavior in intestinal tumorigenesis (121). Correspondingly, loss of mucus would result in increased penetration of epithelial barriers and enhanced microbial translocation through the colon, leading to colorectal neoplasms in mouse models (122).
(2) Proinflammatory molecules—many inflammatory cytokines and growth factors have been reported to facilitate tumor development or antitumor immunity. In mice bearing breast cancer, IL-1 was upregulated and its related signaling was enhanced, which could promote angiogenesis, endothelial cell adhesion, lymphocyte polarization, and recruitment of myeloid cells, thus contributing to cancer progression and bone metastasis (123–125). Suppressed action of IL-1 dramatically inhibited tumor growth in ovarian cancer mouse models (126). IL-17 can promote GI tumorigenesis by binding to its receptor, and this signaling could induce the activation of the mitogen-activated protein kinase (MAPK) and NF-κB pathways and boost colonic epithelial cell proliferation and further support malignant transformation in mice (121, 127). TNF-α exhibited its protumorigenic features through activation of representative c-Jun N-terminal kinase (JNK) and NF-κB signaling pathways, resulting in enhanced epithelial to mesenchymal transition (EMT) and accelerated tumor cell invasion (128, 129).
(3) ECM remodeling—ECM remodeling such as degradation or stiffening was tumorigenic (130). It could contribute to tumor invasion and metastasis, in which integrin clustering could encourage focal adhesions, intensify ERK and PI3K pathways, and thus promote cell proliferation and invasion (131, 132).

Moreover, other mechanisms may also participate in the process of *Malassezia*-related inflammation-cancer transformation, such as DNA lesion accumulation and imbalance of oncogenes and antioncogenes. Inflammatory cells may release cytotoxic chemicals such as reactive oxygen species (RONS) to induce DNA damage (133). Continuous inflammatory conditions may result in aggravation and accumulation of DNA damage in cells, which may promote genetic mutations, generate genomic instability, and eventually cause carcinogenesis (134). Another powerful toxic polycyclic aromatic hydrocarbon, 7,12-dimethylbenz[a] anthracene (DMBA), has been reported to induce inflammation-dependent dermal tumorigenesis in mice through the cGAS-STING signaling pathway (135) and even distant metastasis in mouse models of breast cancer (136). Accumulated mutations in oncogenes such as c-Myc, which may be induced by inflammatory cytokines or DNA damage, could show synergism with inflammatory stimulus to enhance oncogenous process including enhanced cell proliferation, differentiation, and malignant transformation (137, 138). In addition, chronic inflammation may contribute to tumor protein 53 (TP53) mutations in the epithelium, and this accumulation could lead to deep loss of tumor suppressor functionality in cells. As a result, chromosomal instability increased and eventually cancer occurred (139, 140). In turn, the tumor microenvironment may exacerbate tumorigenic inflammation, leading to a persistent vicious circle between inflammation and cancer.

High-abundance *Malassezia* has already been detected in multiple cancers, implying that *Malassezia* may be a key in initiating and accelerating cancer development. Furthermore, the presence of *Malassezia* was inextricably linked with its induced inflammation. Based on this, during the process of *Malassezia* in cancer promotion, inflammation may be the biggest contributor.

## Conclusion

In general, patients with IBD exhibit a high incidence in a series of cancers. For the IBD population, increased level of *Malassezia* is connected to the imbalance of microflora, fungal translocation, and inflammatory deterioration. In addition, the abundance of *Malassezia* is positively correlated with the pathogenesis and progression of various cancers, suggesting that *Malassezia* may be a key component to relate IBD with cancer. Accordingly, broad-spectrum antifungal drugs, aimed to reduce the production of *Malassezia* strains or their metabolites, can inhibit tumorigenesis and slow down tumor progression, by restoring internal fungal homeostasis and reducing inflammatory responses. This may provide new thoughts for cancer monitoring and novel therapeutic approaches. However, more studies are still required to verify the clinical benefits of *Malassezia* genus inhibition in IBD or cancer group. Although *Malassezia* is considered to be a pathogenic fungus which may participate in the pathogenesis of IBD and promote this disease to further develop into cancer, more informative pathogenesis still needs to be addressed. Multidisciplinary cooperation has become an inevitable trend, so the next arduous task is to deeply explore and fully utilize *Malassezia*-relevant mycology, and then combine it with metabolomics and immunology. This future research will reveal the potential of *Malassezia* strains as therapeutic targets, aiming at relieving inflammatory reaction, improving patient outcome in IBD, and further reducing the incidence of cancer.

## Data Availability Statement

The original contributions presented in the study are included in the article/supplementary material. Further inquiries can be directed to the corresponding authors.

## Author Contributions

QY and JO wrote the first draft edition of the manuscript. DP provided critical revisions of this manuscript. LF and JY conceived and designed the manuscript. All authors listed have made a substantial, direct, and intellectual contribution to the work and approved it for publication.

## Funding

Our work was funded by the Joint Medical Research Project (2020GDRC010) of Chongqing Science & Technology Bureau and Chongqing Health Commission and the Chinese Federation of Public Health foundation (GWLM202024).

## Conflict of Interest

The authors declare that the research was conducted in the absence of any commercial or financial relationships that could be construed as a potential conflict of interest.

## Publisher’s Note

All claims expressed in this article are solely those of the authors and do not necessarily represent those of their affiliated organizations, or those of the publisher, the editors and the reviewers. Any product that may be evaluated in this article, or claim that may be made by its manufacturer, is not guaranteed or endorsed by the publisher.
